# Enhanced Chemokine Receptor Expression on Leukocytes of Patients with Alzheimer's Disease

**DOI:** 10.1371/journal.pone.0066664

**Published:** 2013-06-18

**Authors:** David Goldeck, Anis Larbi, Mariavaleria Pellicanó, Iftikhar Alam, Inga Zerr, Christian Schmidt, Tamas Fulop, Graham Pawelec

**Affiliations:** 1 Tübingen Ageing and Tumour Immunology Group, Center for Medical Research, University of Tübingen, Tübingen, Germany; 2 Singapore Immunology Network (SIgN), Agency for Science Technology and Research, Biopolis, Singapore, Singapore; 3 Abdul Wali Khan University, Mardan, Pakistan; 4 Clinical Dementia Center, Dept. of Neurology, University Medical School, Georg August University, Göttingen, Germany; 5 Immunology Program, Geriatric Division, Faculty of Medicine, Research Center on Aging, University of Sherbrooke, Sherbrooke, Quebec, Canada; New York University, United States of America

## Abstract

Although primarily a neurological complaint, systemic inflammation is present in Alzheimer's Disease, with higher than normal levels of proinflammatory cytokines and chemokines in the periphery as well as the brain. A gradient of these factors may enhance recruitment of activated immune cells into the brain via chemotaxis. Here, we investigated the phenotypes of circulating immune cells in AD patients with multi-colour flow cytometry to determine whether their expression of chemokine receptors is consistent with this hypothesis. In this study, we confirmed our previously reported data on the shift of early- to late-differentiated CD4+ T-cells in AD patients. The percentage of cells expressing CD25, a marker of acute T-cell activation, was higher in patients than in age-matched controls, and percentages of CCR6+ cells were elevated. This chemokine receptor is primarily expressed on pro-inflammatory memory cells and Th17 cells. The proportion of cells expressing CCR4 (expressed on Th2 cells) and CCR5 (Th1 cells and dendritic cells) was also greater in patients, and was more pronounced on CD4+ than CD8+ T-cells. These findings allow a more detailed insight into the systemic immune status of patients with Alzheimer's disease and suggest possible novel targets for immune therapy.

## Introduction

The brain of patients with Alzheimer's Disease (AD) is infiltrated by immune cells [1]. T-cells and other immune cells are likely to be present in the brain because they have migrated from the periphery through the blood-brain barrier (BBB) which differs in AD compared to healthy individuals. Under normal physiological conditions, an intact BBB regulates the transport of many of the molecules and cells present in the blood. This is due to the special structure of the endothelium with tight junctions and no pinocytotic activity. The gene expression profile and presence of adhesion molecules is specific for these endothelial cells compared to others within the body [2]. In typical inflammatory diseases, permeability increases and lymphocytes migrate through the BBB into the CNS. The expression of adhesion molecules is also increased [2]–[4]. According to Kovac et al., amyloid beta (Aβ) - the accumulation of which in the brain is a hallmark of AD - increases microvascular permeability [5]. Accordingly, not only T-cells but also monocytes and B-cells migrate through the BBB into the brain. One important role of the B-cells is likely to be the regulation of CNS inflammation via IL-10 production as described by Fillatreau and colleagues [6].

Small molecules such as Aβ cross the BBB [7]–[9]. A link between Aβ and T-cell migration was suggested by Li et al., who showed that Aβ - transported via advanced glycation end products - activates a signaling cascade which leads to CCR5 expression in brain endothelial cells. CCR5 binds to its ligand MIP-1α on T-cells and enhances their mobility [10]. CXCR2 is another chemokine receptor which contributes to regulating T-cell migration. When this receptor is blocked on T-cells (e.g. by antibodies) migration through a BBB model in vitro is inhibited. In contrast, Aβ upregulated CXCR2 expression resulted in enhanced T-cell mobility (in rats). In a second approach, Liu and coworkers investigated the influence of the inflammatory environment by using an anti-TNF antibody; this resulted in inhibition of the CXCR2 ligand IL-8 in human brain microvascular endothelial cells, and further lowered T-cell migration [11]. Several other monocyte chemoattractant proteins (MCPs) and their receptors such as CCL2 and CCR2 are also expressed during neurodegenerative diseases and associated with inflammation and neuronal death [12]. Corrêa et al. found higher CCL2 levels in the CSF of AD patients compared to controls [13].

The impact of Aβ on cell migration is also highlighted in a study by Fiala and co-workers, who showed that peripheral monocytes stimulated with Aβ 1–42 secreted proinflammatory cytokines (TNF, IL-6, IL-1β and IL-12) as well as chemokines (CC chemokines MCP-1, MIP-1α, and MIP-1β, and CXC chemokine IL-8). In an in vitro BBB model, Aβ 1–42 contributed to the increased migration of monocytes through the barrier into the brain side and further to the site of Aβ plaques [14].

These findings prompted the present study, on the basis that the inflammation in AD patients is not restricted to the brain but is systemic and chemokines may play an essential role. Additionally, for the diagnosis of AD, the blood is easier to access than the brain, which is essentially only possible post-mortem, and furthermore it allows follow-up studies on the same patients to evaluate disease progression and eventual treatment efficacy. Hence, the present study focuses on PBMC of AD patients and healthy age-matched controls. The expression of several chemokine receptors (CCR2, 4, 5 and 6) was studied to investigate - reciprocal to their impact on the BBB described above - also the corresponding expression of these molecules on blood leukocytes. Because only memory T-cells but not naïve T-cells appear to be able to migrate into the brain [15], we phenotyped peripheral T-cells in patients and controls to establish which cells expressed chemokine receptors and to what degree. The ultimate aim of this work was to define characteristic peripheral immune signatures that might qualify as biomarkers as well as starting points for novel targeted treatments, because modulating the migration of leukocytes might facilitate disease control.

## Materials and Methods

### Ethics statement

Informed, written consents were provided by the patients. In case the patients were not able to comprehend the implications of a study participation and thereby were unable to consent due to the extent of their cognitive impairment as assessed by the study physician, the legal caregivers were asked to provide the written consent. The study has been performed in accordance to the Declaration of Helsinki. The local ethics committee has approved the study (Georg August University, Göttingen, Germany, votum No 9/6/08).

### Patients and Controls

Well-characterised AD patients considered for inclusion had to have a diagnosis of probable AD consistent with the Diagnostic and Statistical Manual of Mental Disorders - Fourth Edition (DSM-IV) as set out by the American Psychiatric Association in 1994, and the Dubois criteria [16] and were in the same diagnostic range. A CT or MRI scan was required to corroborate the AD diagnosis. Major Stroke or massive vascular lesions on imaging were exclusion criteria. All patients were free of any other chronic inflammatory diseases. Patients (12 female, 11 male; mean age: 69±9) with other forms of dementia as e.g. Parkinson's disease, vascular dementia, mixed dementia or pseudodementia were excluded from this study. Healthy elderly people acted as controls (11 female, 9 male; mean age: 68±3).

### Sample collection and surface marker staining

A method was established and validated for the analysis of frozen blood [17]. Briefly, 10% DMSO was added directly to blood samples, which were frozen by placing them in a −80°C refrigerator. Samples were thawed by placing them in a water bath at 37°C until ice clumps were just still visible. Samples were diluted 1∶25 with buffer (PBS, 2 mM EDTA, 2% FBS) and centrifuged for 5 min at 300× g to remove DMSO.

Cells were equilibrated in PFEA staining buffer (PBS, 2% FCS, 2 mM EDTA and 0.01% Na azide). All steps were performed at room temperature. After centrifugation for 5 min at 300× g the pellet was resuspended in 50 µl 1% Gamunex ® (human immunoglobulin; Bayer, Leverkusen, Germany) for blocking and 1 µg EMA for live-vs-dead cell discrimination. After 10 min incubation (+10 min under light for EMA staining) 1 ml PFEA was added for washing, and cells spun down (300× g, 5 min). A cocktail of conjugated antibodies was then added (samples protected from light; antibodies titrated for PBMC in 50 µl). The reaction volume as made up to 50 µl with PFEA. In parallel, the same amount of antibody was added to a compensation control with BD comp beads. For each antibody, a separate control was prepared. For each antibody mouse or rat beads were used according to the isotype and also negative beads from the same batch to visualize positive and negative signals. Samples and controls were incubated in the dark for 30 min with regular mixing. Cells were resuspended in 200–300 µl PFEA and acquisition started on an LSR II with FACSDiva 6.1 software (BD Biosciences). For analysis FlowJo 7.2.5 software was used (TreeStar, Ashland, OR).

The following antibodies were used in this study: CCR2-PerCP-Cy5.5, CCR5-FITC, CCR6-Pacific blue, CD45RO-Alexa Fluor 700 (BioLegend, San Diego, CA, USA); CCR4-PE-Cy7, CD3-Alexa Fluor 700, CD4-PerCP, CD8-PerCP, CD25-V450, CD25-APC-Cy7, CD28-PerCP-Cy5.5, CD45-V500, PD-1-PE (BD Biosciences); CD3-Pacific Orange, CD19-Qdot605, CD14-Qdot644, CD27-Qdot605, CD45RA-Qdot655, CD8-Qdot705 (Invitrogen, Karlsruhe, Germany); CD4-APC (Miltenyi Biotec, Bergisch Gladbach, Germany).

All statistical analyses were performed with Graphpad Prism 4.02 (2004) or JMP 8.0 (SAS Institute, 2008). Non-parametric Mann-Whitney U test was used for comparison of independent groups with Bonferroni correction.

## Results

### T-cell phenotypes

**Figure 1:**
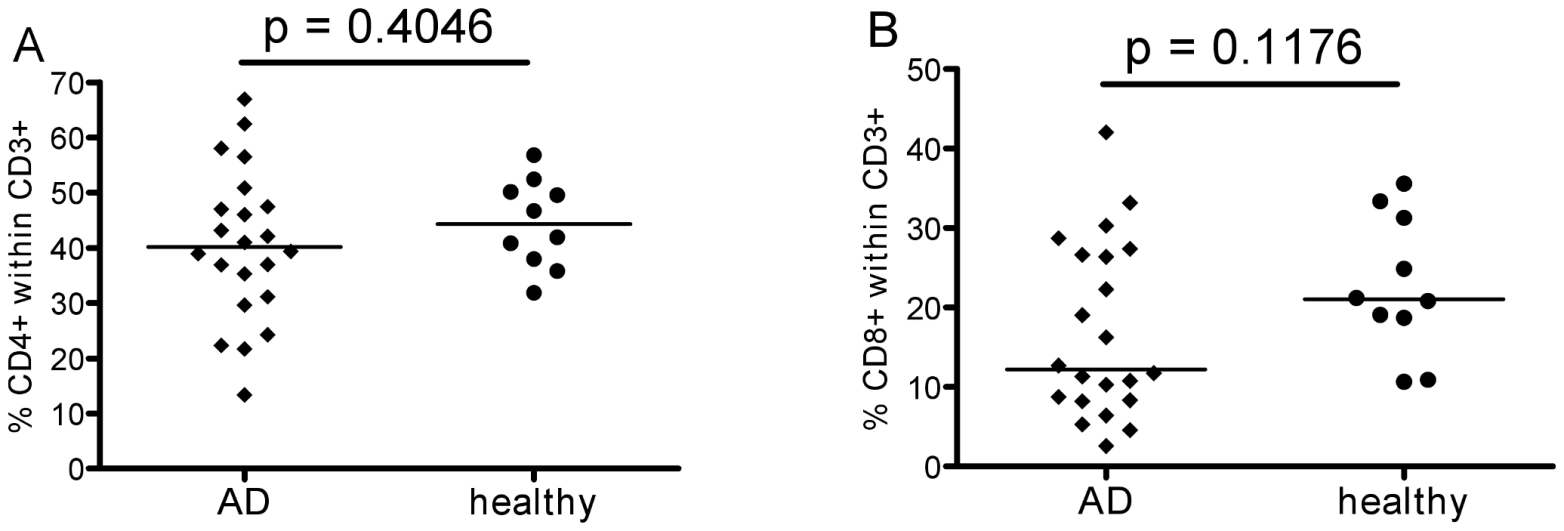
T-cell subsets. Percentages of CD4+ (A) and CD8+ (B) T-cell subsets. After selecting living single CD3+ T-cells, the populations of CD4+ and CD8+ cells were gated. The values of 23 AD patients are displayed in diamonds and compared to those of 10 healthy controls in circles.

**Figure 2:**
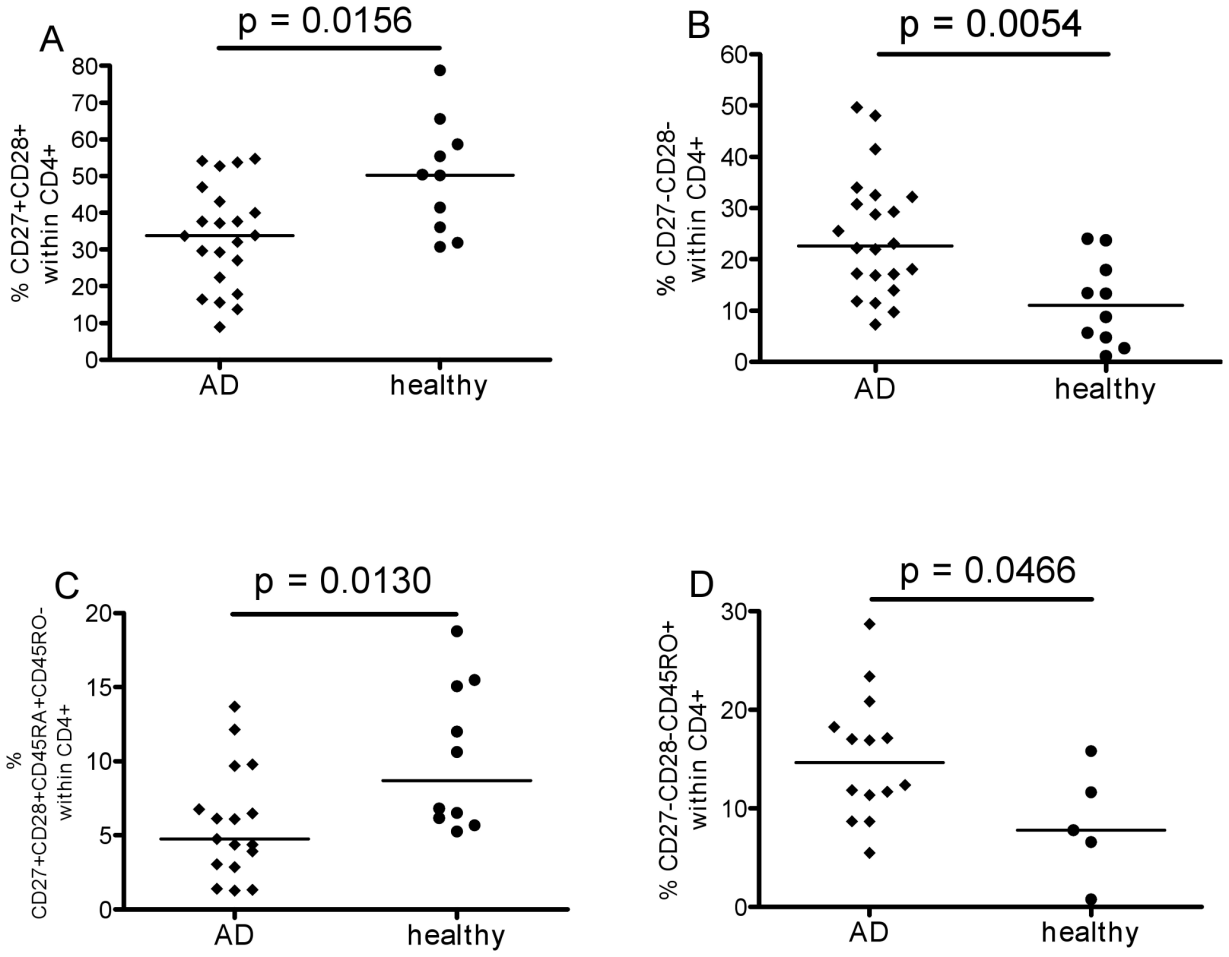
Naïve and memory CD4+ T-cells. Percentages of early-differentiated CD4+ T-cells in A (CD27+CD28+) and C (CD27+CD28+CD45RA+CD45RO-); percentages of late-differentiated T-cells in B (CD27-CD28-) and D (CD27-CD28-CD45RO+). For statistical analysis Mann Whitney test was applied and resulting p values are displayed.

As assessed in peripheral blood, people generally accumulate memory T-cells, especially in the CD8+ compartment, and have fewer naïve T cells, due to increasing antigen exposure with time over trhe lifespan. To investigate whether this situation was in any way different in AD, T-cell phenotypes were analysed and also T-cell homing capacity as reflected in chemokine receptor expression. Percentages of CD4+ and CD8+ cells within the CD3+ T-cell population did not differ between patients and controls (Figure 1), and there were no significant differences in the CD4∶CD8 ratio (data not shown). However, a more detailed analysis of the T-cell phenotypes did reveal certain differences. The expression of the costimulatory receptors CD27 and CD28 on CD4+ T-cells (Figure 2A and 2B) was significantly different between patients and controls. The percentage of early-differentiated CD27+ CD28+ cells was lower in AD samples compared to healthy controls and the percentage of late-differentiated CD27- CD28- cells was greater. Consistent results were obtained using additional markers for classifying T-cell differentiation stages. Thus, naïve cells characterized as CD27+CD28+CD45RA+CD45RO- were more frequent in healthy controls and late memory cells - defined as CD27-CD28-CD45RO+ - were less frequent (Figure 2C and 2D).

### Chemokine and cytokine receptor expression

**Figure 3:**
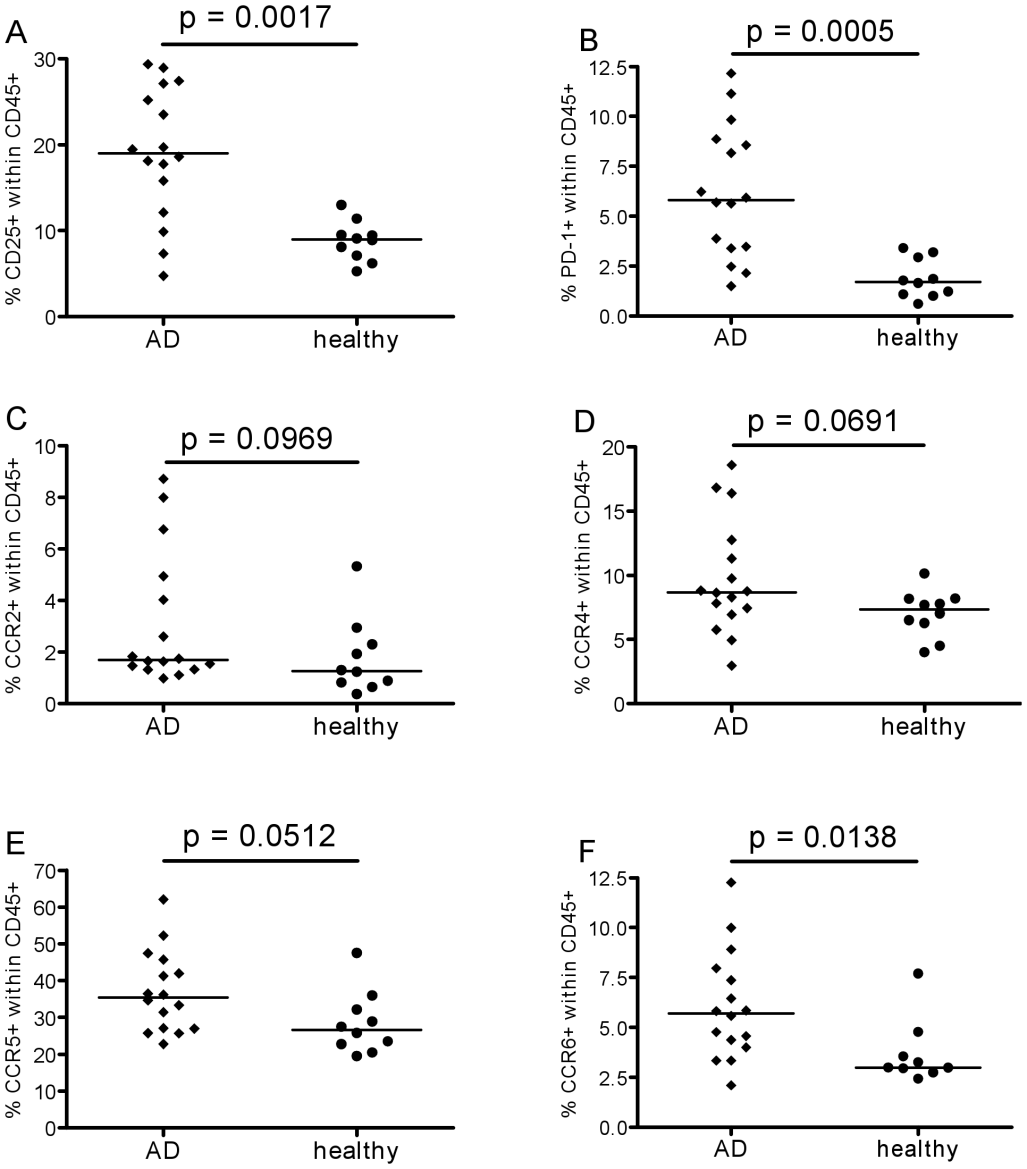
Leukocyte phenotypes. Percentages of activated CD25+ (A), exhausted PD-1+ (B), CCR2+ (C) (mainly expressed on activated memory cells), CCR4+ (D), CCR5+ (E) and CCR6+ (F) cells within CD45+ total leukocytes of 16 AD patients (diamonds) and 10 healthy controls (circles). The lines represent the median.

First, activated leukocytes expressing the alpha chain of the IL-2 receptor, CD25, were analysed. A higher proportion of CD25+ cells within the CD45+ leukocytes was found in patients compared to age-matched controls, suggesting a more activated status. A putative marker of T cell exhaustion, the PD-1 receptor, was also more frequently expressed by AD patients' cells (Figure 3B).

**Figure 4:**
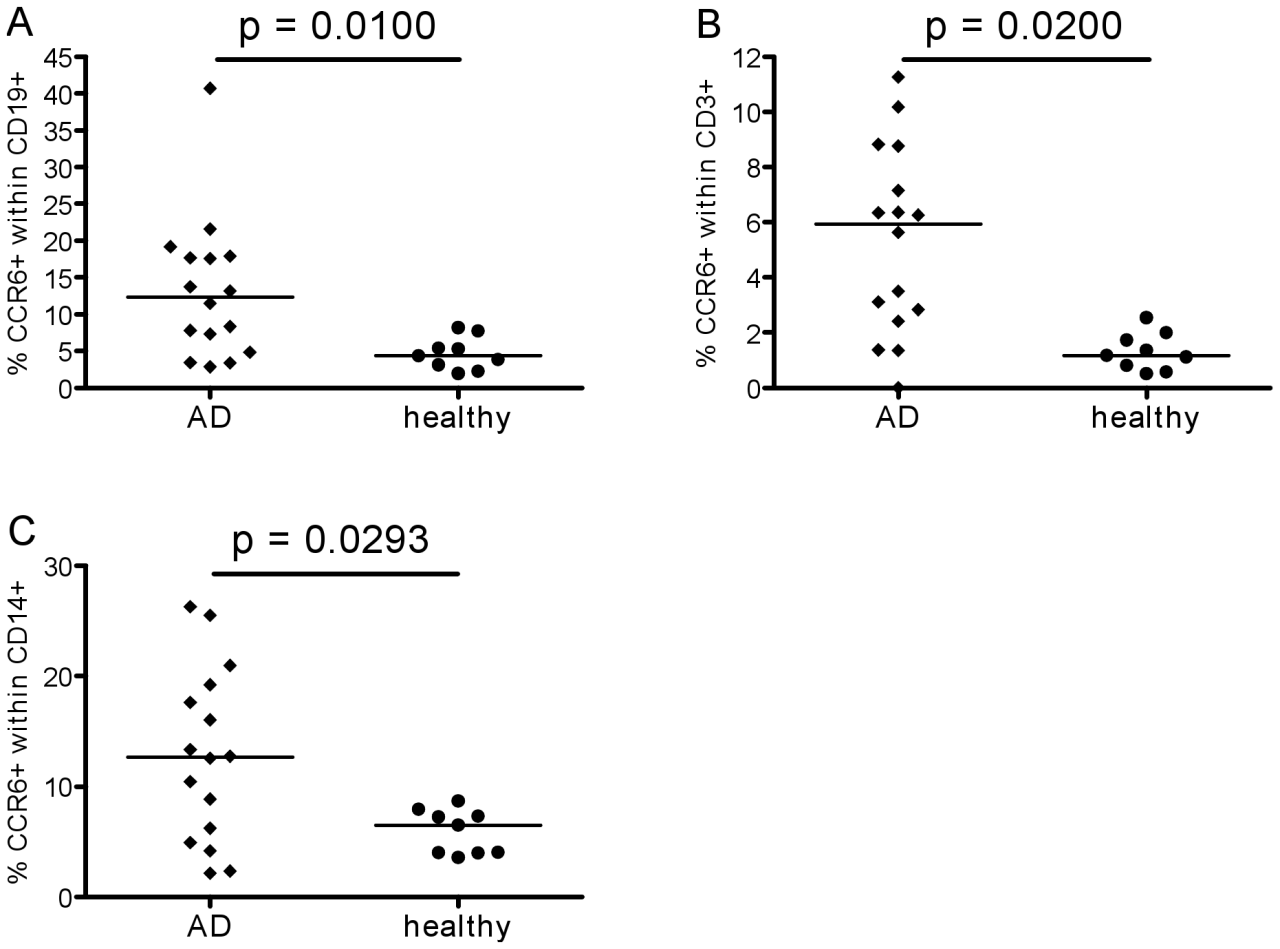
CCR6 expression on leukocyte subsets. CCR6 expression on different leukokocyte (CD45+) populations: percentages of CCR6+ B-cells (CD19+) (A), T-cells (CD3+) (B) and monocytes (CD14+) (C) after surface marker staining with different antibodies as described in the material and methods section.

**Figure 5:**
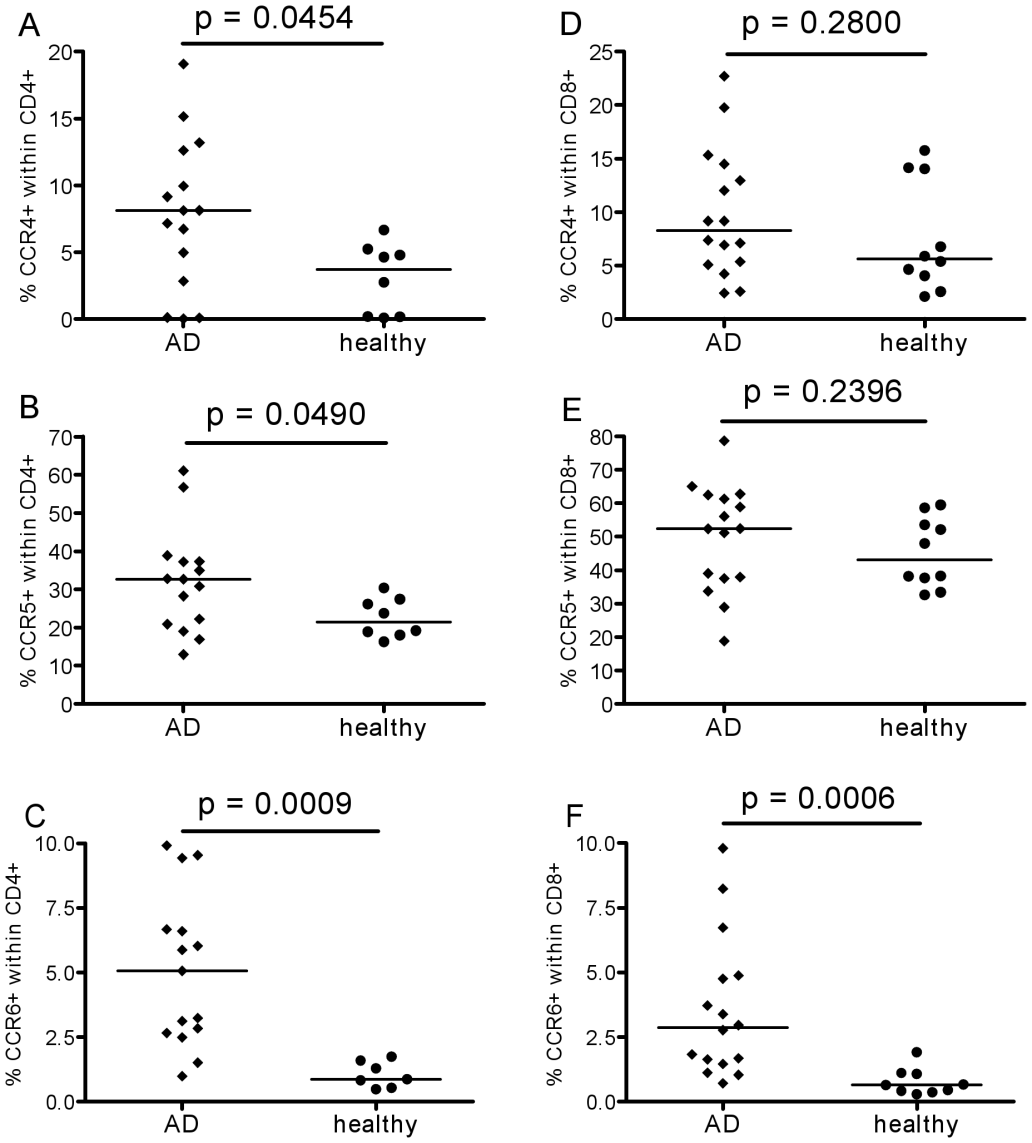
Chemokine receptor expression on T-cell subsets. Expression of the chemokine receptors CCR4 (on Th2 cells), CCR5 (on differentiated Th1 cells) and CCR6 (mostly on non activate memory cells and Th17 cells) on CD4+ (left-hand panels) and CD8+ T-cells (right-hand panels) in AD patients (diamonds) and healthy controls (circles) with medians and p values obtained by Mann Whitney analysis using Graphpad Prism.

Next, chemokine receptor expression was investigated. First, results on total CD45+ leukocytes are shown in Figures 3C–F. The expression of CCR2 and CCR4 was not different in the two groups, although some individual AD patients had higher frequencies of positive cells than any of the controls. The percentages of cells expressing CCR5 tended to be greater in AD patients than healthy elderly (Figure 3E). In contrast, percentages of CCR6+ cells were significantly greater in AD patients (Figure 3F). A more detailed analysis was therefore performed to investigate which leukocytes were expressing higher levels of this chemokine receptor (Figure 4). Within the total leukocyte population, monocytes (CD14+), B-cells (CD19+) and T-cells (CD3+) were gated separately. It was found that CCR6 expression was higher in AD patients than controls on all three cell types. Further analysis of chemokine receptor expression on T-cells revealed that the percentages of CCR4+ and CCR5+ cells within the CD4+ subset were greater in AD, albeit only just achieving significance (Figure 5A, B), whereas the difference in CCR6 expression was highly significant (Figure 5C). The difference in CCR6 expression between AD and controls on CD8+ T cells was also highly significant (Figure 5F), whereas this was not the case for CCR4 and CCR5 (Figure 5D, E).

## Discussion

Although primarily a neurological disease, systemic inflammation is present in AD, a disease with higher than normal levels of proinflammatory cytokines and chemokines in the periphery as well as in the brain [18] as reviewed by Lee et al. [19]. T-cells can be stimulated by pro-inflammatory mediators - due to the characteristic systemic inflammation in AD - as well as by various other agents, possibly including amyloid beta (Aβ) [20]. As a result, immune cell differentiation may be enhanced. One example shown here is the low percentage of early-differentiated CD4+ T-cells accompanied by a higher percentage of late-differentiated CD4+ T-cells. These findings confirm our earlier results obtained with different antibody staining models used to analyse T-cell phenotypes of AD patients from different countries (Canada and Italy). This suggests the robustness of this finding across cohorts and techniques. Also in those cohorts, the percentages of memory CD4+ T-cells were higher in AD patients compared to healthy controls [21], [22]. A shift from early to late-differentiated T-cells can be observed in other circumstances where chronic antigen exposure is thought to be prevalent, for example in a study of young and elderly blood donors from Pakistan [23]. These findings from different populations with different environment, genetic background and lifestyle, suggest a general outcome of long-term antigen exposures. Together with higher PD-1 and CD25 expression in AD patients, these results imply that the adaptive immune system in AD patients is in a more activated state than controls and may be becoming “exhausted” possibly due to Aβ chronic antigenic stimulation, and/or other presently unidentified stressors. It is of note that these differences are discernible in the blood, despite the fact that the main damage in AD occurs in the brain. The data presented in the current study may provide a link between immune status in the periphery and the brain.

In AD, immune cells migrate along a chemokine gradient from the periphery through the blood-brain barrier into the brain [14]. Hence, the present study focused on chemokine receptor expression on PBMC. Reale and others postulated that chemokine receptors are expressed in the brain of healthy individuals and AD patients, but that several of them are more up-regulated in the latter [18]. The dysregulation of chemokines and their receptors is postulated to lead to an accumulation of microglia in senile plaques and may play a role in a cascade which ends with neuronal death. Increased concentrations of cytokines and chemokines in AD are reported in both the periphery and brain but it is still debated which is the driving force and which a consequence of the other. Genetic analyses have shown that expression of CCR2, CCR5 and CCL5/RANTES is increased while CCL2/MCP-1 is decreased in AD. This increase was also observed when PBMC were stimulated in vitro with Aβ-42 [18]. Another study showed elevated CCR2 and CCR5 expression on T-cells and as well on B-cells after stimulation of PBMC from AD patients and healthy controls with Aβ-42. In the case of CCR5, T-cells of AD patients expressed more than controls [24]. Lee and colleagues used CCR5 knockout mice and found elevated Aβ-42 levels and higher beta-secretase expression in the brain compared to wild-type mice [25]. The authors hypothesized that missing CCR5 leads to an activation of CCR2 which through astrocyte activation in turn leads to Aβ deposition and memory dysfunction [25]. The CCR5 signaling pathway also influences the expression of other chemokine receptors such that in CD4+ T-cells of CCR5 knockout mice expression of CCR1, CCR2 and CXCR3 is reduced, with consequences for CNS trafficking [26]. Therefore, CCR5 might be a crucial suppressor of the development and progression of AD pathology and the observed higher expression on leukocytes of AD patients a possible mechanism to control the disease. Further support for this hypothesis is the possible neuroprotective role of CCR5 [27]. Thus, it can be suggested that our data showing tendential increases of CCR5 expression on PBMC may reflect attempts to counteract the pro-inflammatory nature of other CCRs such as CCR6.

It is also believed that CCR6 may play an important role in this context. Together with adhesion molecules like selectins and integrins, CCR6 influences T-cell migration through the choroid plexus into the CSF [28], e.g. the recruitment of Th17 cells is CCR6-CCL20 mediated [29]. IL-17 cells might cause anegative effects on neurons, tissue and the BBB. Experiments with mice show higher CCR6 expression in brain and periphery of transgenic mice with AD-like disease, which the authors suggested was due to the systemic inflammation in AD [30]. Our present findings in humans are similar to those in mice regarding the inflammatory nature of AD.

A direct influence of CCR2 on disease progression was shown by El Khoury and collaborators [31]. In mice lacking CCR2 they observed less microglial accumulation, less Aβ clearance and more promotion of early disease progression [31]. That especially Ly-6C(hi)CCR2+ monocytes are those which are recruited to brain lesions and which then differentiate into microglia was published by Mildner and colleagues using a mouse model [32]. Regarding CCR2 ligands, Westin et al. found a correlation between CCL2 levels in the CSF and more rapid cognitive decline in MCI patients with prodromal AD on follow-up [33].

In our study, we analysed CCRs from a different point of view. We investigated the expression on PBMC and were able to confirm the previous results of Reale and coworkers. Additionally, we included other CCRs in the analysis and investigated CCR expression on different leukocytes as well. We found that CCR2, CCR4, CCR5 and CCR6 were expressed at slightly higher levels on the entire CD45+ population of AD patients compared to controls, but AD associations were stronger when leukocyte subsets were analyzed separately. Notably, CCR6 expression was higher in all the main cell types, T-cells (CD3+), B-cells (CD19+) and monocytes (CD14+). Within the T-cell subset, higher CCR4 and CCR5 expression was seen on CD4+ than CD8+ cells, in line with observed phenotype changes that are mainly focused on the CD4+ compartment in AD [21], [22]. Significantly, and in agreement with the results of Pellicanó and colleagues in Italian patients [21], greater production of the CCR5 ligand MIP-1α by T-cells - as reported previously [34] – together with the expression of CCR5 by human brain microvascular endothelial cells, suggest a mechanism supporting T-cell migration across the BBB.

One hypothesis is that certain immune cells which infiltrate the brain contribute to inflammation; thus, controlling trafficking might help to decrease the inflammation. Additionally, recruited microglia might secrete neurotoxins causing neurodegeneration [31], which could be reduced by preventing cells from entering the brain. An advantage of surveying several chemokine receptors is that different leukocytes do not express all receptors at the same time, but only certain patterns of receptors, so that one might be able to specifically avoid targeting those with a beneficial role in controlling the disease. B-cells might help to control CNS inflammation through IL-10 secretion [6] and can also release nerve growth factors that promote regeneration [35]. Aβ activates neuroprotective microglia via a signaling cascade starting with the scavenger receptor CD36 and recruits them to amyloid plaques [36]. Aβ can be phagocytosed by macrophages and thereby reduce the negative effects of Aβ. Experiments from Zaghi et al. showed that *“AD macrophages ingested and cleared less, and underwent apoptosis upon exposure to soluble, protofibrillar, or fibrillar Aβ”* [37]. Another study of Fiala et al. showed that in contrast to those from controls, monocytes from the blood of AD patients take up Aβ but mediate insufficient clearance, have less capacity for differentiation into macrophages, and more readily undergo apoptosis [38]. In transgenic mice, experiments with GFP-labelled Aβ showed that blood-derived monocytes enter the brain and phagocytose Aβ [27], [39], [40]. On the other hand, these results are controversial, due to the nature of the model; some might be artifactual and not represent the in vivo situation (reviewed by Cameron et al., ref. [41]). However, our data showed increased expression of CCR6 in monocytes and B cells suggesting that they may have important regulatory roles in the brain when recruited there.

Our results confirm the important changes in chemokine receptors on PBMCs of AD patients compared to controls, potentially linking these inflammatory cells to brain damage. Our study cannot determine whether these changes are beneficial or harmful for the development or the progression of the AD. This awaits follow-up studies of these patients,which are in progress. It can be speculated that the specific interactions between the various immune cells may be beneficial at the beginning and harmful in later stages, as shown in several experiments cited above (6, 31, 35, 37). In future experiments it would be interesting to investigate whether blocking or enhancing migration of various peripheral leukocytes into the brain provides advantages in controlling inflammation, amyloid plaques and immune exhaustion, or whether it might have adverse effects. The present study highlights possible new therapeutic target candidates based on biomarkers more highly expressed by peripheral blood cells from AD patients than controls. Town et al. reviewed the role of T-cells in AD and highlighted the likely beneficial effects of Th2 T-cells in murine models. In AD patients, one reason that T-cells fail to exert such beneficial effects to control disease may be the high NO production around amyloid plaques, resulting in T-cell apoptosis (20). Therefore it could be advantageous to specifically promote chemotaxis to increase brain infiltration by anti-inflammatory peripheral T-cells. On the other hand decreeasing migration of inflammatory T-cells with e.g. antibodies directed against certain chemokines or their receptors, such as CCR6, could reduce the inflammation present in AD.
